# Study the Use of Activated Carbon and Bone Char on the Performance of Gravity Sand-Bag Water Filter

**DOI:** 10.3390/membranes11110868

**Published:** 2021-11-11

**Authors:** Eric Fung, Ken I. Johnson, Wenqi Li, William Borges, Kai Chi, Sunil K. Sharma, Yogita Madan, Priyanka R. Sharma, Benjamin S. Hsiao

**Affiliations:** 1 Department of Chemistry, Stony Brook University, Stony Brook, New York, NY 11794-3400, USA; eric.fung@stonybrook.edu (E.F.); ken.johnson@stonybrook.edu (K.I.J.); wenqi.li7@gmail.com (W.L.); William.borges@brown.edu (W.B.); jacknjfu@gmail.com (K.C.); sk5040@gmail.com (S.K.S.); 2 Center for Integrated Electric Energy Systems, Stony Brook University, Stony Brook, New York, NY 11794-6044, USA; 3 Department of Chemistry, Amity School of Applied Sciences, Jaipur 303002, India; madan.runa@gmail.com

**Keywords:** granulated activated charcoal, bio charcoal, biosand filter, Langmuir Isotherm, adsorption

## Abstract

In this study, granulated activated charcoal (GAC) and bio charcoal (BC) is used as a filler in P3 biosand bag filter to study their filtration performance against a range of fluoride impurities from 1–1400 mg/L. A set of experiments are done to analyze the filtration efficiency of the sandbag filter against fluoride impurities after incorporating different amounts (e.g., 0.2, 2 kg) and a combination of GAC and BC. A combination of filler GAC and BC (1 kg each) have exhibited excellent results with 100% fluoride removal efficiency against 5 mg/L fluoride impurities for an entire experimental time of 165 min. It is because of the synergetic effect of adsorption caused by the high surface area (739 m^2^/g) of GAC and hydroxyapatite groups in BC. The data from remediation experiments using individual GAC and BC are fitted into the Langmuir and Freundlich Isotherm Models to check their adsorption mechanism and determine GAC and BC’s maximum adsorption capacity (*Q_m_*). The remediation data for both GAC and BC have shown the better fitting to the Langmuir Isotherm Model with a high R^2^ value of 0.994 and 0.970, respectively, showing the excellent conformity with monolayer adsorption. While the GAC and BC have presented negative Kf values of −1.08 and −0.72, respectively, for Freundlich Model, showing the non-conformity to multilayer adsorption. The *Q_m_* values obtained from Langmuir Model for GAC is 6.23 mg/g, and for BC, it is 9.13 mg/g. The pH study on adsorption efficiency of individual GAC and BC against 5 mg/L of fluoride impurities indicates the decrease in removal efficiency with an increase in pH from 3 to 9. For example, BC has shown removal efficiency of 99.8% at pH 3 and 99.5% at pH 9, while GAC has exhibited removal efficiency of 96.1% at pH 3 and 95.9% at pH 9. Importantly, this study presents the significance of the synergetic application of GAC and BC in the filters, where GAC and BC are different in their origin, functionalities, and surface characteristics.

## 1. Introduction

One major issue affecting the health and hygiene of people all over the world is the lack of clean water. According to news releases from UNICEF and the WHO, about 2.2 billion (one in three) people globally do not have access to safe drinking water and billions of others are left without proper washing facilities. If water is not made more affordable and accessible to all, it is estimated that half of the global population in 2025 will live in water-stressed conditions [1]. Currently, this is also largely concerning during the COVID-19 pandemic because 1.8 billion people who use or work in healthcare are at a higher risk of infection due to the lack of access to basic water services [2].

Water can be contaminated by several types of impurities. Most common are heavy metals impurities which are usually present in their charged forms such as cations and anions. They are widespread and can cause environmental toxicity in addition to a multitude of acute and chronic illnesses depending on the amount ingested over time. For example, lead is one heavily researched heavy metal in drinking systems that can cause nervous system, respiratory system, and multiple organ-related damages. Among non-metal impurities, fluoride is an example of a potential anion contaminant in water. There is natural hydrogeological dissolution and distribution near marine sediments or volcanoes. However, waste dumping by industrial smelting, semiconductor, and other manufacturing industries also releases fluoride. While beneficial at around 0.7–1.2 mg/L in drinking water, it can cause dental fluorosis above 2 mg/L and skeletal fluorosis above four mg/L. Because of this, there are estimates that perhaps millions have endemic fluorosis due to bioaccumulation [1]. Many novel alumina, rare earth elements, and silica-gel based adsorbents have been determined to have high adsorption capacities for fluoride, but most of these are too expensive to be widely used [3]. Nanocellulose prepared from cost-effective method such as the nitro-oxidation method, is also known as an excellent adsorbent for water purification [4,5,6,7,8,9,10,11,12,13,14,15,16]. The World Health Organization (WHO) also claims excess fluoride removal is expensive and that the best method is to just find safe water, but that is often not possible.

A biosand filter is a traditional gravity filtration technique that has been used to remove pathogens and suspended solids from water using different adsorbents through physical and biological processes that occur in sand columns covered with a biofilm. One of the examples of biosand filters is the P3 biosand bag filter [17]. The concept behind the biosand filter is a mix of slow sand filtration and biofilm formation due to naturally found aquatic organisms. More specifically, the Schmutzdecke biofilm that takes around two weeks to form on the top of the sand layer, is a gelatinous matrix and contains bacteria, fungi, protozoa. It has been established that these microorganisms form microcolonies and biofilm as a survival strategy and activate extracellular polymeric substances as protection from pH, salts, any mechanical or chemical stressor, and even antibiotics [18]. This means this mucilaginous film can effectively trap foreign particles and adsorb the dissolved organic matters and metabolized them. Furthermore, it has been documented that certain biofilm can even attach to acid mine drainage substrates such as heavy metals but under specific conditions such as pH [19]. These types of biosand filters can be assembled by using sand, charcoal, activated charcoal, or any other adsorbent.

In general, the activated charcoal is formed when biomass undergoes physical activation by pyrolysis in an inert atmosphere. Occasionally, the charcoal is activated by post-treatments using specific acids, bases, salts, or peroxides. These activation processes strip away the residual particles resulting in a highly porous carbon material with basic aromatic, oxygen, and nitrogen functional groups. The surface area of activated charcoal can reach up to around 2000 m^2^/g. Additionally, its surface contains carboxylic and other functional groups, which allow the successful removal of heavy metal ions [20]. Activated charcoal has been found to be a cost-effective method for water filtration. While activated carbons are excellent for heavy metals, they are usually lacking in anion adsorption capabilities. However, the bone char which contains the hydroxyapatite functionality can adsorb the fluoride impurities via ion exchange and electrostatic interaction [3].

In this study, we have used the commercially available granular activated charcoal and bone char as a filler for the P3 sandbag filter. The well-managed P3 biosand bag filter made of the sand column has been determined to remove over 90–99% of bacteria and viruses, while it has been never demonstrated as an effective and reliable filter for other chemical contaminants such as heavy metals and anion contaminants. Here, we have first time demonstrated the P3 filter comprised of GAC and BC as an effective water filter against fluoride impurities. Our aim is to demonstrate the utility of the P3 sandbag filter to not only tackle the biological contaminants but also to limit the fluoride contaminants in the water.

## 2. Materials and Methods

### 2.1. Materials

Commercially available GAC from hardwood having a size of 8 × 16 mesh and BC (mixed animal bone) with the size of 8 × 24 mesh was obtained from Charcoal House LLC (Crawford, NE, USA). These materials are traditionally used for air and liquid filtration applications. For testing, samples were repeatedly washed with deionized water to remove residual powders. Finally, these were used to filter the simulated sodium fluoride (NaF) contaminated water. Varying concentrations of fluoride solution from 5 to 2620 mg/L were prepared for remediation experiments. For GAC sample, 5–580 mg/L of fluoride impurities were tested, and fitted their data into Langmuir and Freundlich isotherm model. While for BC, 50–2620 mg/L of fluoride impurities were chosen, and their data were plotted into Langmuir and Freundlich isotherm model. The amount of GAC and BC used to do the static adsorption study was 0.2 g. However, the amount of GAC and BC used for the sandbag filtration unit is presented in Section 2.4 and Section 3.5.

### 2.2. Methods

#### 2.2.1. Fourier Transform Infra-Red Spectrometry (FTIR)

FTIR (Nicolet IS50 from Thermo Scientific, Waltham, MA, USA) was performed on the crushed sample to determine the functional components of both the GAC and BC. FTIR curves were recorded in the transmission mode, between 450 and 4000 cm^−1^. A total of 6 scans were taken per sample with a resolution of 4 cm^−1^. The solid samples were recorded in the Attenuated Total Reflectance (ATR) mode.

#### 2.2.2. Brunauer-Emmett-Teller (BET) 

BET analysis (Quantachrome NOVAtouch LX2 Analyzer from Quantachrome Instruments, Boynton Beach, FL, USA) was used to determine pore size distribution and adsorption graphs. For degassing, 0.15 g charcoal was heated at 100 °C for 12 h at 10 °C/min. Surface analysis was done at full isotherm with 9 mm rod for pellet samples.

#### 2.2.3. Zeta Potential Measurement

The surface charge of samples was measured using a ZetaProbe (ZetaProbe from Colloidal Dynamics, Ponte Vedra Beach, FL, USA). A 150 g of powdered samples were dispersed in distilled water to make the concentration of 1.81 wt%. A total of 10 measurements were taken per analysis with no delay.

### 2.3. Static Adsorption Study

To determine maximum adsorption capacity (*Q_m_*), static adsorption was performed with a 0.2 g sample at varying concentrations of fluoride (10 mL). Fluoride levels were measured using an ion selective electrode (HACH HQ30D, Hach Company, Loveland, CO, USA) and modeled using the Langmuir and Freundlich adsorption isotherms with the following expression:

Langmuir Adsorption Isotherm Model Equation:

(1) $$\frac{C_{e}}{Q_{e}}=\frac{C_{e}}{Q_{m}}+\frac{1}{Q_{m}b}$$

where *C_e_* is equilibrium concentration of adsorbate F^−^, *Q_e_* is the experimental adsorption capacity of F^−^ at equilibrium, and *Q_m_* to be calculated by plotting *C_e_/Q_e_* vs. *C_e_* using the Equation (1), *Q_m_* was determined by the inverse of the slope.

Freundlich Adsorption Isotherm Model Equation:

(2) $$\mathrm{lg}Q_{e}=\frac{1}{n}\mathrm{lg}C_{e}+\mathrm{lg}K_{F}$$

where: *C_e_*—equilibrium concentration of adsorbate F^−^, *Q_e_* is the experimental adsorption capacity of F^−^ at equilibrium^−^; *K_F_* and n are the characteristic constants of the system.

### 2.4. Biosand Bag Filtration Unit

**Figure 1:**
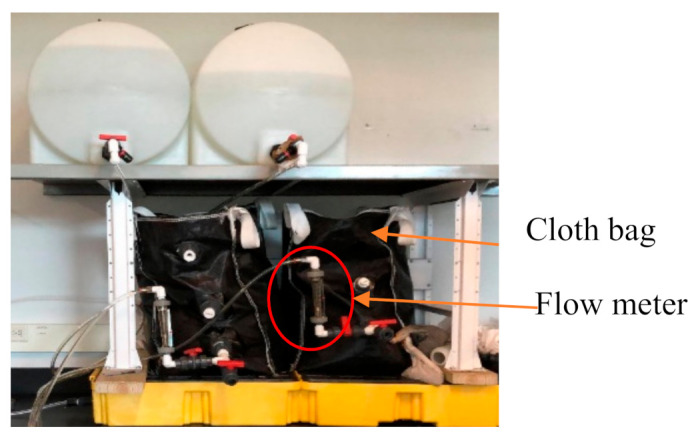
Biosand bag filtration setup.

A 381 mm× 381 mm × 635 mm scale-down sample bag was obtained from biosand bag LLC. A total of 45.3 kg 0.5 mm silica sand was set and cleaned with bleach and 132.4 L tanks were used to flow simulated contaminated water with 5 mg/L fluoride into the filter bags. The biosand filtration setup is presented in Figure 1.

Flow meters were attached to simulated water tanks to adjust the flow rate. The effluent release was kept between 0.4–0.8 LPM measured using flow meters while the water level in the bag was kept between 100–150 mm. Varying amounts of GAC and BC were placed in cloth bags above the sand and 132.4 L of simulated contaminated water was run through the filtration system to determine removal efficiency.

## 3. Results and Discussion

### 3.1. FTIR Analysis of GAC and BC

**Figure 2:**
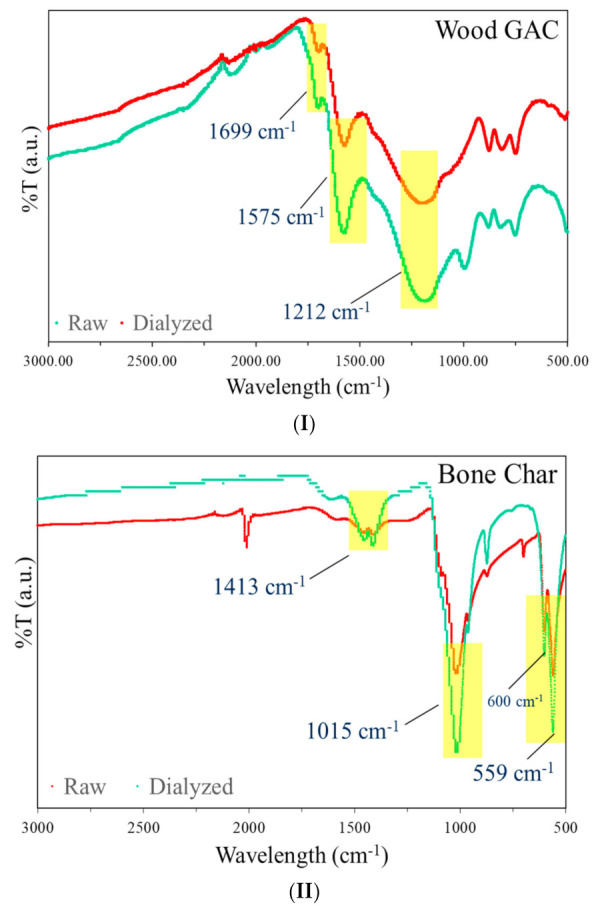
Fourier transform infrared (FTIR) spectra of (**I**) raw and dialyzed granulated activated charcoal (GAC), (**II**) raw and dialyzed bone char (BC).

FTIR spectra of the GAC and BC were performed to analyze their functional groups are presented in Figure 2. The FTIR spectra of hardwood GAC showed the following major peaks: (I) 1698 cm^−1^ indicates the presence of C=O stretching shows the presence of conjugated ketones, (II) 1574 cm^−1^ peak relates to C=C stretching of aromatic components and lignin, (III) 1211 cm^−1^ indicate hydrogen-bonded phosphorous functional group linkages [21]. These peaks remained unchanged after dialysis, indicates that hardwood GAC as such contains no residual impurities.

While the FTIR of BC presented the following characteristic peaks: (I) 871 cm^−1^ demonstrates the presence of carbonate groups in hydroxyapatite structure, (II) a broad and strong band of calcium (Ca^2+^) was at 560 and 610 cm^−1^ indicates the structure of calcium hydroxyapatite, (III) 1050 cm^−1^ stretching mode of phosphate (PO_4_^3−^) peak. These bands are characteristics of mineral calcium hydroxyapatite [Ca_5_(PO_4_)_3_(OH)] which is the main constituent of teeth and bones [22]. These characteristic peaks remain maintained in dialyzed BC, however, the minor peaks at 1413 and ~800 cm^−1^ disappeared in BC after dialysis. These peaks probably belong to residual degraded protein and fats that may have attached to bones even after pyrolysis and washing. FTIR characterizations determine that the most important functional groups are present in the samples. Hardwood GAC contains C=O, C-O, and aromatic C-C stretches, and BC possesses C-O and hydroxyapatite groups.

### 3.2. Surface Area Analysis

The surface area is a critical property that describes the adsorbent surface characteristics. The BET is the most common method used to measure the surface area of adsorbent using N_2_ gas as an adsorbate onto the surface of a material. The results presented in Table 1 reveals that the measuring surface area of the GAC is 739 m^2^/g while the BC showed a surface area of 74 m^2^/g. The total pore volume of the hardwood GAC was determined to be 0.237 cm^3^/g while the total pore volume of the BC was measured to be 0.86 cm^3^/g for pores less than a radius of 69 nm. The average pore size for hardwood GAC was 2.3 nm and for BC was 6.3 nm. The characteristic of the charcoal is highly dependent on the source and method of preparation.

BET surface analysis results were consistent with other studies on activated carbon and bone char. Surface area, total pore volume, and average nanopore diameter were all within the dimensions resulting from activating acorn shells at varying temperatures [23]. Bone char was only compared to Fija Fluor bone char from another study, but the results were very similar. BC had slightly less surface area, but more pore volume and a smaller average nanopore size.

### 3.3. Zeta Potential Measurement

Zeta potential is related to the net surface charge that the substrates have. Hardwood GAC was acidic (pH = 4.2) in suspension and has a negative surface charge of −2.4 mV consistent with potential carboxylate surface groups as indicated by 1699 cm^−1^ peak in FTIR. The BC was basic (pH = 8.1) in suspension and possessed a positive surface charge of 4.5 mV consistent with the hydroxyapatite groups. The results demonstrate that the BC can attract the negatively charged impurities (e.g., Fluoride) more efficiently than the BC substrate.

### 3.4. Langmuir and Freundlich Adsorption Isotherm Models

**Figure 3:**
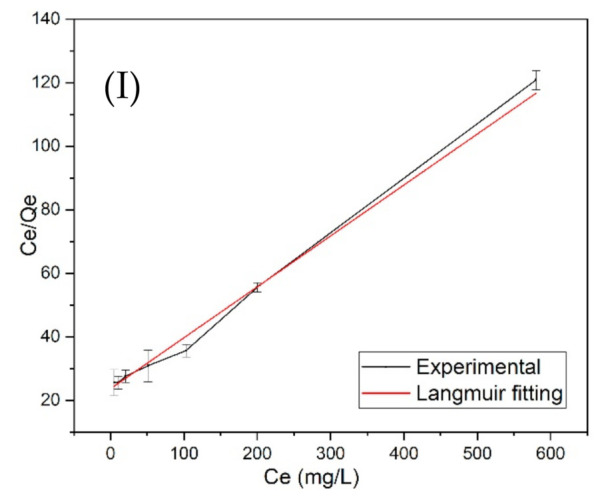

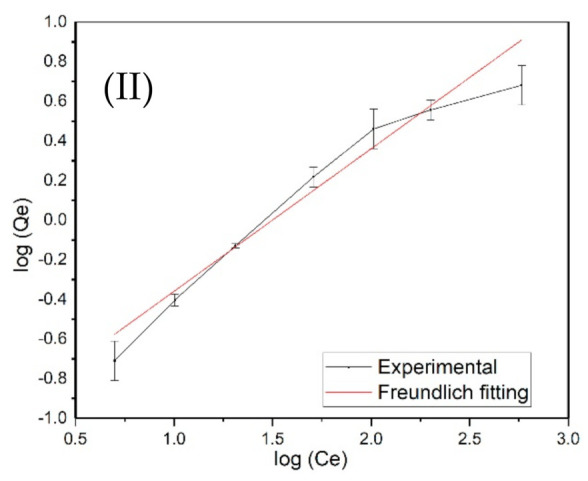
(**I**) Langmuir Isotherm plot and (**II**) Freundlich isotherm plot for fluoride adsorption by hardwood granular activated carbon (GAC).

**Figure 4:**
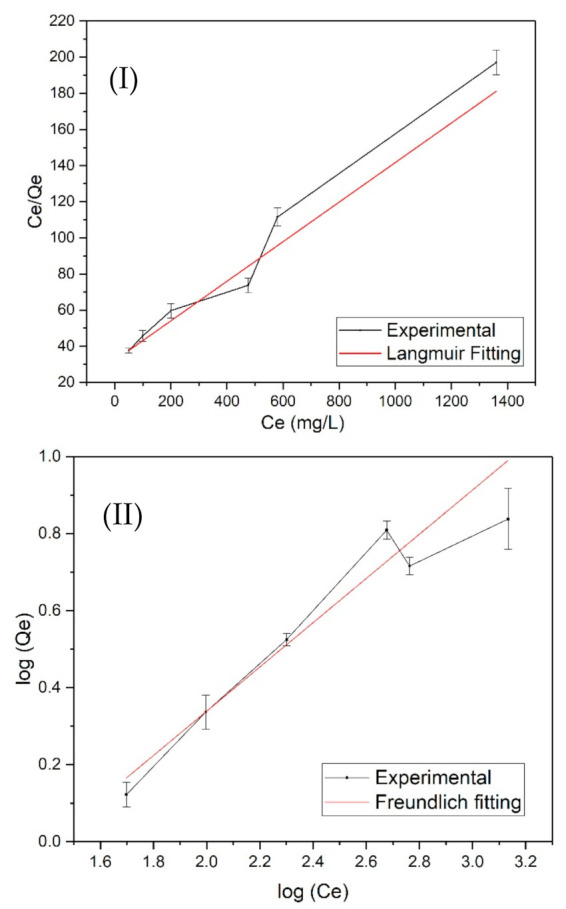
(**I**) Langmuir Isotherm plot and (**II**) Freundlich isotherm plot for fluoride adsorption by bone char (BC).

The Langmuir model is an ideal monolayer adsorption model that assumes that all the adsorption sites have equivalent adsorption energies and that there are no mutual interactions between adsorbed molecules. *Q_m_* determined from the Langmuir Isotherm Model (Figure 3I) for hardwood GAC was 6.23 mg/g. The data showed excellent Langmuir fitting with an R^2^ value of 0.9946. Similarly, BC showed *Q_m_* of 9.13 mg/g with an R^2^ value of 0.970 (Figure 4I). The results indicate that the Langmuir model can better describe GAC and BC adsorption behavior, suggesting that the adsorption process tended to be monolayer adsorption. The data was also applied in the Freundlich Isotherm Model, and the plot of logQe versus logCe is exhibited in Figure 3II and Figure 4II. The summary of adsorption model parameters is presented in Table 2. The results indicate that the GAC and BC better fit the Langmuir Isotherm Model than Freundlich Model. It could be because of the heterogeneity of the GAC and BC layer that has prevented the better molecules interactions. Moreover, the R^2^ values for both GAC and BC with Freundlich Isotherm are slightly different from R^2^ values obtained in Langmuir fittings. However, the Kf values obtained were negative, which shows the non-fitting of GAC and BC remediation data to the Freundlich model, which expresses multi-layer adsorption.

Langmuir adsorption isotherm (Figure 3 and Figure 4) determines that BC indeed had a higher maximum adsorption capacity than the hardwood GAC. However, the GAC and BC in this study have shown the *Q_m_* slightly lower than the reported adsorbents shown in Table 3. For example: activated charcoal of *Catha edulis* showed the removal efficiency of 18 mg/g with a removal efficiency of 73% at the optimum conditions of adsorbent dose of 1.5 g in 100 mL and contact time of 60 min and pH 2 [24]. Activated carbon obtained from *Vitex negundo* also showed 50–99% removal efficiency when 1–12 mg/g of fluoride impurities were tested [25]. Similarly, lapsi seeds presented 50–99% removal efficiency against 1–25 mg/L of fluoride impurities. However, *Crocus sativus* and modified sludge presented the removal efficiency in the range of 80–85% when 1–10 mg/L fluoride impurities were tested [26]. A vast literature has been reported on the use of bone char for fluoride removal [27,28,29,30]. The presence of hydroxyapatite groups in bone char is effective in removing the fluoride impurities by ion exchange and electrostatic interaction mechanism. For example, Bone char from cattle can adsorb ~11 mg/g of fluoride at pH 3 when 20 mg/L of fluoride impurities are present.

**Figure 5:**
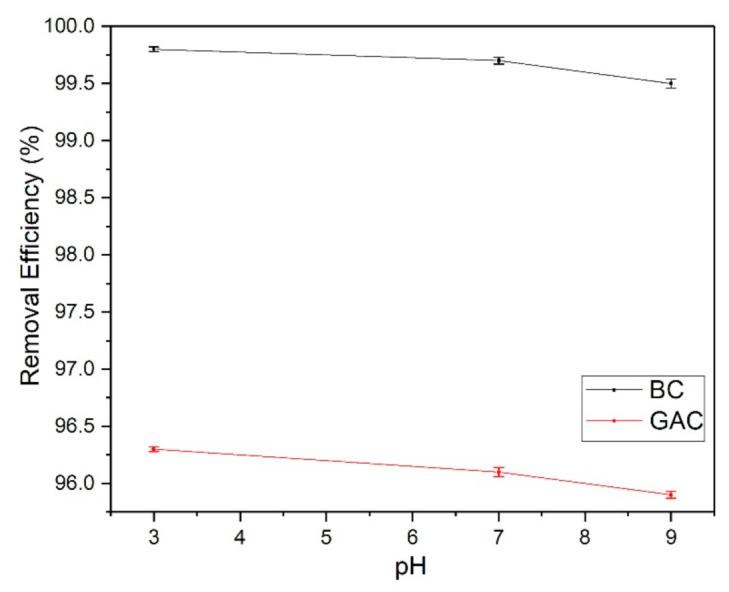
Effect of pH change in adsorption of fluoride (5 mg/L) by granular activated carbon (GAC) and bone char (BC) separately.

In this study, a wide range of fluoride concentrations have been tested against GAC and BC samples. For GAC, we have tested 1–600 mg/L of fluoride impurities and for BC we have tested 1–1400 mg/L of impurities. It was observed that the GAC sample reached its maximum removal capacity quickly at 600 mg/L while the BC sample took much longer to reach its maximum removal capacity around 1400 mg/L. This is because BC is enriched with hydroxyapatite active groups responsible for fluoride trapping, and GAC lacks hydroxyapatite, but it possesses other active groups such as carboxyl groups. The lower *Q_m_* value of GAC and BC samples than other adsorbents is probably due to their testing on a wide range of fluoride impurities. The effect of pH on fluoride adsorption with GAC and BC samples is shown in Figure 5. This study was performed at three different pH (pH 3, 7, and 9). The results indicate that BC’s fluoride removal capacity was higher in the range of 99.5–99.8% than the GAC samples with the efficiency of 95.9–96.1% because of the presence of highly active functional groups hydroxyapatite on BC. However, both GAC and BC have shown a decrease in removal efficiency with an increase in pH. The reduction in the removal of fluoride efficiency with an increase in the pH is probably due to the competition between hydroxyl groups and fluoride ions in an alkaline environment.

### 3.5. Sand-Bag Gravity Filtration

**Figure 6:**
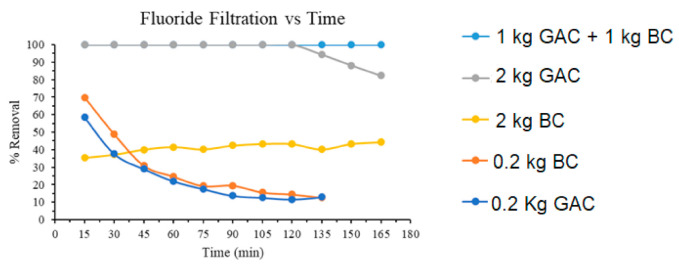
Percent fluoride removal by varying amounts of granular activated carbon (GAC) and bone char (BC).

Interesting results were observed when GAC and BC samples when incorporated in a biosand bag filter and tested for fluoride removal. Different sets of samples were used to check the performance of sandbag filtration units. These setups were: (I) GAC-0.2 kg, (II) GAC-2 kg, (III) BC-0.2 kg, (IV) BC-2 kg, (V) Mixer of GAC-1 kg and BC-1 kg. The filtration results are shown in Figure 6 where the different sets of samples were tested against 132.4 L of 5 mg/L of fluoride simulated contaminated water. The use of a low amount (0.2 kg) of adsorbents, GAC, and BC has shown a rapid decrease in fluoride impurities starting with the removal of 60 and 70% respectively and reached the lower capacity of 10–20% quickly only after 60 min of the run. However, increasing the amount of GAC and BC to 2 kg increased the final removal rate to 100 and 35% respectively. The removal efficiency dropped down to 80% after two hour runs. This is most probably due to saturation of pores and active sites due to trapping of Fluoride impurities. While BC showed the constant removal efficiency of ~35% even after the 165 min run. BC possesses the hydroxyapatite groups to trap the fluoride impurities, but it possessed surprisingly low percent removal perhaps due to its lower surface area and larger pore size than GAC. However, a combination of GAC-1 kg and BC-1 kg demonstrated excellent results with 100% removal for the entire 132.4 L water tank for 165 min. A combination of both hardwood GAC and mixed BC yielded the best results over the entire experiment, potentially due to synergistic effects. BC most probably interacted with fluoride impurities via electrostatic interaction caused in between its hydroxyapatite groups and negatively charged fluoride impurities, while GAC due to high surface area may have shown excellent adsorption due to high internal area for adsorbate and their good van der Val forces of interaction.

## 4. Conclusions

This study shows that the combination of GAC and BC demonstrated the excellent removal efficiency of ~100% and long operation durability in the sandbag filter against fluoride impurities. A combination of factors such as surface charge, surface area, pore size, pore-volume, and the substrate behavior in different pH, all affect the performance of the charcoal-like substrate. For example, GAC has shown the better removal of fluoride impurities than BC, even though the GAC possesses the negative surface charge of −2.4 mV, but the surface area of GAC was 739 m^2^/g which is several times higher than the BC substrate with the surface area of 74 m^2^/g. And this could be the reason that GAC showed excellent removal efficiency for fluoride impurities via adsorption. Additionally, a combination of GAC and BC showed the excellent adsorbent because of their synergetic effects on excellent removal of fluoride ions via electrostatic interaction via BC and Adsorption by GAC. This gravity filtration using activated carbon and bone char is proven to be very efficient and easy to set up. Low cost and easy transportation also would allow for feasible distribution to water-stressed areas around the world.

## Figures and Tables

**Table 1:** Surface area and surface charge comparisons.

	Surface Area (m^2^/g)	Total Pore Volume (cm^3^/g)	Average Pore Diameter (nm)	Surface Charge (mV)	Reference
Harwood GAC	739.0	0.237	2.321	−2.9	This work
Mixed BC	74.9	0.86	6.32	4.5	This work
Fija Fluor BC	104	0.30	11.1	NA	[3]

**Table 2:** Summary of adsorption isotherm model parameters.

Adsorbent	Langmuir Model			Freundlich Model		
	*Q_m_* (mg/g)	*K*	R^2^	*K_F_*	*n*	R^2^
GAC	6.23	0.16036	0.9946	−1.0822	1.430	0.997
BC	9.13	0.10949	0.970	−0.7228	1.896	0.9664

**Table 3:** Comparison of GAC and BC with other relevant adsorbents reported in the literature.

Adsorbents	Maximum Adsorption Capacity (mg/g)	Removal Efficiency%/ Fluoride Concentration (mg/L)	References
Activated charcoal of *Catha edulis*	18	80–85/1–10	[24]
Activated carbon of *Vitex negundo*	1.02	99–50/1–12	[25]
Activated carbon of *Lapsi seeds*	-	50–99/1–25	[31]
*Crocus sativus* leaves	-	85/6.5	[26]
Bark of *Morinda tinctoria*	26	-	[32]
Modified sludge adsorbent	1.5	81/1–5	[33]
Cattle BC	11.9	-	[1]
GAC	6.23	100/5	This work
BC	9.13	40/5	This work
GAC + BC	-	100/5	This work

## Data Availability

The data presented in this study are available on request from the corresponding author. The data are not publicly available due to their relevance as part of an ongoing projects.
